# Transgender persons receiving gender-affirming hormone therapy: risk of acute cardiovascular events in a Dutch cohort study

**DOI:** 10.1093/eurheartj/ehaf837

**Published:** 2025-11-04

**Authors:** Lieve Mees van Zijverden, Abel Thijs, Jeske Joanna Katarina van Diemen, Chantal Maria Wiepjes, Martin den Heijer

**Affiliations:** Department of Internal Medicine, Amsterdam University Medical Centre, De Boelelaan 1117, 1081HV Amsterdam, The Netherlands; Centre of Expertise on Gender Dysphoria, Amsterdam University Medical Centre, De Boelelaan 1117, 1081HV Amsterdam, The Netherlands; Amsterdam Public Health, Amsterdam University Medical Centre, De Boelelaan 1117, 1081HV Amsterdam, The Netherlands; Department of Internal Medicine, Amsterdam University Medical Centre, De Boelelaan 1117, 1081HV Amsterdam, The Netherlands; Department of Internal Medicine, Amsterdam University Medical Centre, De Boelelaan 1117, 1081HV Amsterdam, The Netherlands; Department of Internal Medicine, Amsterdam University Medical Centre, De Boelelaan 1117, 1081HV Amsterdam, The Netherlands; Centre of Expertise on Gender Dysphoria, Amsterdam University Medical Centre, De Boelelaan 1117, 1081HV Amsterdam, The Netherlands; Amsterdam Public Health, Amsterdam University Medical Centre, De Boelelaan 1117, 1081HV Amsterdam, The Netherlands; Department of Internal Medicine, Amsterdam University Medical Centre, De Boelelaan 1117, 1081HV Amsterdam, The Netherlands; Centre of Expertise on Gender Dysphoria, Amsterdam University Medical Centre, De Boelelaan 1117, 1081HV Amsterdam, The Netherlands; Amsterdam Public Health, Amsterdam University Medical Centre, De Boelelaan 1117, 1081HV Amsterdam, The Netherlands

**Keywords:** Transgender, Gender-affirming hormone therapy, Cardiovascular events, Myocardial infarction, Stroke, Thrombosis

## Abstract

**Background and Aims:**

Despite favourable effects of oestradiol on cardiovascular risk factors, previous studies found higher risks of cardiovascular events in transgender women using gender-affirming hormone therapy. However, these studies did not adjust for socioeconomic status and lifestyle. This study investigated the association between gender-affirming hormone therapy and cardiovascular event risk in a large cohort of transgender persons compared with the general population, accounting for these factors.

**Methods:**

This retrospective cohort study included transgender women and transgender men using gender-affirming hormone therapy between 1972 and 2018 at the Amsterdam gender clinic. Medical diagnoses were registered from 2012 to 2022 by a national data registry. Standardized incidence ratios for myocardial infarction, ischaemic cerebrovascular accident, and venous thromboembolism were computed using general population incidence rates adjusted for socioeconomic status, estimated by education, employment, and income. Lifestyle (body mass index, smoking, and alcohol consumption) was analysed by age group.

**Results:**

Adjusted for socioeconomic status, transgender women (*N* = 2714, 23 907 person-years) had lower myocardial infarction risk (.50 [.32; .71]), similar cerebrovascular accident risk (.94 [.72; 1.19]), and higher venous thromboembolism risk (1.81 [1.33; 2.35]) compared with general population men. Transgender men (*N* = 1617, 13 457 person-years) had higher myocardial infarction risk (4.20 [2.72; 6.01]), higher cerebrovascular accident risk (1.55 [1.01; 2.20]), and similar venous thromboembolism risk (1.00 [.53; 1.61]) compared with general population women. Socioeconomic status minimally impacted these results. Lifestyle largely resembled the general population.

**Conclusions:**

Gender-affirming hormone therapy is not associated with increased cardiovascular risk in transgender women but is in transgender men. This aligns with known effects of oestradiol and testosterone on cardiovascular risk factors.

## Structured Graphical Abstract

**Structured Graphical Abstract:**
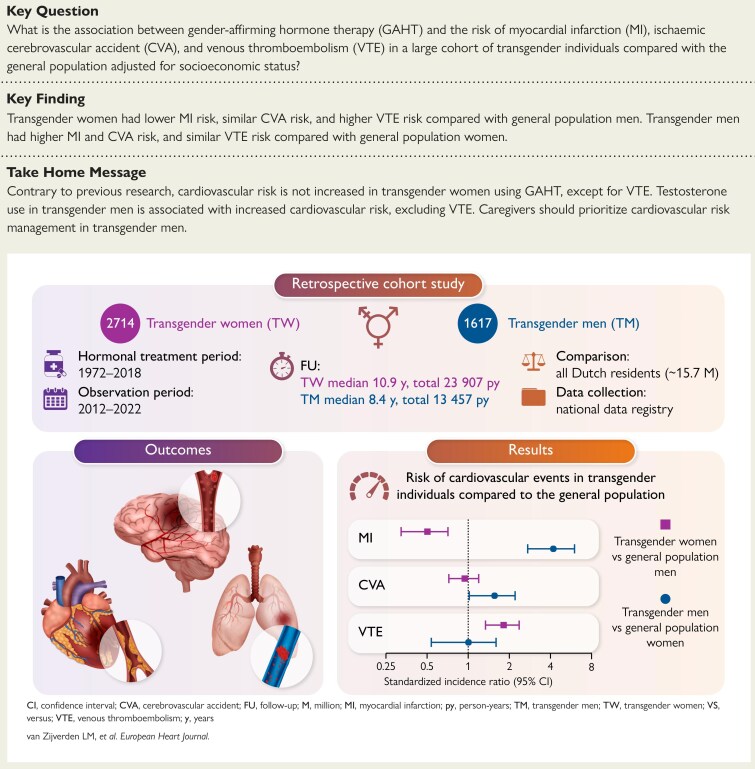
Risk of cardiovascular events in transgender individuals compared with the general population, adjusted for socioeconomic status.

**See the editorial comment for this article ‘Walking the tightrope: balancing safety and risk in gender-affirming hormone therapy’, by A. Kharlamov, https://doi.org/10.1093/eurheartj/ehaf939.**

## Introduction

Men generally have a higher cardiovascular risk than women, which is partially attributable to differences in sex hormones. In women in the general population, endogenous oestradiol appears to protect against arterial cardiovascular disease.^1,2^ A protective effect of oestradiol would therefore also be expected in transgender women. However, previous studies consistently reported a discrepancy in the expected cardiovascular disease pattern in transgender persons: both transgender men (assigned female sex at birth, male gender identity) and transgender women (assigned male sex at birth, female gender identity) using gender-affirming hormone therapy (GAHT) have an increased risk of cardiovascular events.^3,4^ Several factors have been investigated to understand the elevated risk in transgender women, but no explanation has been identified, as cardiovascular risk factors in transgender people were consistent with those in cisgender people: testosterone use in transgender men is associated with adverse cardiometabolic changes, including decreased HDL and increased LDL and blood pressure. Oestradiol use in transgender women has demonstrated a favourable effect on these parameters.^5–7^ Thus, until now, the striking paradox remains unresolved.

However, previous studies on cardiovascular events in transgender people exhibit several methodological limitations that might explain these inconsistent findings. These limitations include small cohorts, short follow-up periods, loss to follow-up, inappropriate selection of control populations, cross-sectional study designs, and reliance on self-reported data.^4^ Furthermore, socioeconomic status (SES) and lifestyle factors are mostly not accounted for. Socioeconomic status is a well-established determinant of health, and low SES is associated with adverse health outcomes and reduced life expectancy.^8,9^ Transgender individuals generally experience lower SES compared with their cisgender peers, as reflected in disparities in income, social standing, and legal conditions.^10–12^ Additionally, discrimination and stigma faced by transgender persons can negatively impact their health and socioeconomic well-being.^13,14^ It is also universally recognized that lifestyle factors, particularly obesity and smoking, have a detrimental impact on cardiovascular health. Despite this, the potential impact of SES and lifestyle is frequently overlooked, risking the misattribution of increased cardiovascular risk solely to GAHT.

We investigated the risk of myocardial infarction (MI), ischaemic cerebrovascular accident (CVA), and venous thromboembolism (VTE) in our large cohort of transgender persons receiving GAHT, compared with incidence rates in men and women from the general population. By using an actuarial life table approach, stratified by age, sex, and calendar year, while adjusting for SES and exploring potential lifestyle differences, we aim to overcome the limitations of previous studies and to fill a significant gap in the current literature on the long-term cardiovascular safety of GAHT in transgender persons.

## Methods

### Study design and participants

This retrospective cohort study used data from the Amsterdam Cohort of Gender dysphoria (ACOG) from the Centre of Expertise on Gender Dysphoria (CEGD) at the Amsterdam University Medical Centre, The Netherlands. The ACOG includes all persons who visited the CEGD from its establishment in 1972 to 2018. Inclusion criteria for this study are starting GAHT before 31 December 2018. Exclusion criteria are starting GAHT before age 17, prior use of puberty suppression, alternating use of oestradiol and testosterone, having no follow-up visit after initiating GAHT, inability to be linked with the national data registry Statistics Netherlands (The Hague, the Netherlands), or death before the start of the observation period. The national data registry Statistics Netherlands collects and provides, among other metrics, demographics and medical data for all residents of the Netherlands. This enabled comparisons between transgender individuals and women and men in the general population. The reference group in our study consisted of all individuals residing in the Netherlands between 2012 and 2022 (15.7 million people, born from 1917 to 2004). As this was a retrospective cohort study, no sample size calculation was performed. The study protocol was approved by the Medical Ethics Committee of the Amsterdam University Medical Centre. The Medical Research Involving Human Subjects Act does not apply to this study and the necessity for informed consent was waived.

Different types of hormone modalities were used within the ACOG, as hormone therapy protocols have changed over time. Feminizing GAHT consists of oestradiol, mostly accompanied by anti-androgens. Oestradiol was prescribed as ethinyl oestradiol (EE) (25–100 mcg daily), conjugated oestrogens (.625–1.25 mg daily), oestradiol valerate or hemihydrate (2–6 mg daily), injections (10–100 mg every 2–4 weeks), implants (20 mg every 3–6 months), patches (50–150 µg/24 h), gel (.75–3 mg daily), or spray (3–6 mg daily). From 2001 onwards, mostly oestradiol valerate, hemihydrate, patches, gel, and spray were used. Anti-androgens were prescribed as cyproterone acetate (10–100 mg daily), spironolactone (100–200 mg daily), or triptorelin injections (3.75 mg every 4 weeks or 11.25 mg every 12 weeks). From 2018 onwards, mostly triptorelin was used. Masculinizing GAHT consists of testosterone, which was prescribed as gel (20–100 mg daily), intramuscular testosterone esters (125–250 mg every 2–3 weeks), intramuscular testosterone undecanoate (1000 mg every 10–14 weeks), and oral testosterone undecanoate (40–160 mg daily). Oral testosterone was largely phased out from 2006.

### Procedures

For the transgender cohort, transition type, start date of hormone therapy, and dates of follow-up visits were obtained from medical records. Subsequently, the cohort was linked to the nationwide data registry Statistics Netherlands (The Hague, the Netherlands) to obtain data on cardiovascular events, SES, and lifestyle factors. These data were also obtained from Statistics Netherlands for all women and men from the general population, as well as annual population counts on 1 January of each year from 2012 to 2022. The outcomes of interest are the standardized incidence ratios (SIRs) for three major cardiovascular events (MI, CVA, and VTE) in transgender people relative to people with the same and opposite birth sex from the Dutch general population.

#### Cardiovascular events

Statistics Netherlands has registered all hospital-diagnosed cardiovascular events using specific disease codes (diagnosis treatment combination codes) since 2012. Therefore, the observation period for cardiovascular event occurrence in this study runs from 1 January 2012 to 31 December 2022. Cause of death data was not included due to inaccuracy of cardiovascular mortality reporting on death certificates.^15^

#### Socioeconomic status

An overall continuous score for SES for each inhabitant of the Netherlands was determined by Statistics Netherlands based on education level, recent employment history, and household income. These data are recalculated yearly and are available from 2014. For each individual, an average total SES score over the available years was calculated. Thereafter, SES was categorized into three groups (low, middle, high) based on tertiles.

#### Lifestyle

Data on body mass index (BMI; kg/m^2^), smoking history (never/ever), and alcohol consumption (units/week) were collected by Community Health Services via self-reporting in nationwide sample questionnaires in the years 2012, 2016, 2020, and 2022.^16,17^ When data from more than one survey per person were available, a mean BMI, overall smoking status, and mean number of alcoholic units per week were determined before performing the descriptive analyses.

### Statistical analysis

All analyses were performed using Stata statistical software, version 16.1 (StataCorp LLC, College Station, TX, USA). Transgender women and transgender men were analysed separately and compared with general population men and women. Continuous variables were presented as median with interquartile range (IQR). Non-numeric data were presented as absolute numbers and percentage of total. For each outcome, the number of events was presented along with the incidence rate per 1000 person-years with exact Poisson 95% confidence intervals (CIs).

#### Risk of cardiovascular events and adjusting for socioeconomic status

Standardized incidence ratios with 95% CI for MI, CVA, and VTE were calculated using an actuarial life table approach as described by Dickman *et al*.^18^ First, general population incidence rates stratified by age (in 1-year intervals), sex, and calendar year were calculated for each outcome separately, taking only the first event per cardiovascular event type into consideration. Subsequently, the expected number of events in our cohort was calculated by multiplying the age-specific and sex-specific incidence rates of the general population by the number of individuals within each corresponding age and gender group in our cohort. This calculation was performed twice for both transgender women and men: once using birth-assigned males and once using birth-assigned females as reference. Standardized incidence ratios were then calculated by dividing the number of observed cases by the expected cases. The observation period runs from 1 January 2012 to 31 December 2022. Transgender persons were at risk from the start of hormone therapy; however, incidence rates could only be determined from 2012 to 2022. The actuarial life table approach accounts for this by specifying delayed study entry from 2012 to account only for the years in which an individual was effectively at risk. Entry into the observation period is set on 1 January 2012 or on the start date of hormone therapy when this was started at a later date. Exit date was set at cardiovascular event occurrence, at death, or at the end of the observation period. The 95% CI was calculated using the inverse χ2 exact Poisson approach.^19^ Corresponding survival plots were truncated when only 10% of the cohort was still at risk.^20^ The analyses were repeated with SES categorized into tertiles as additional stratification factor. These analyses included 92.9% of transgender women and 96.7% of transgender men, as SES data were available since 2014.

#### Lifestyle

Because lifestyle data were not available for all transgender persons and controls, adjusting for lifestyle was not possible. To still explore their potential effect on expected cardiovascular risk differences, lifestyle factors were analysed descriptively for all groups, stratified by age categories (18–39 years, 40–59 years, and 60+ years). Distribution of BMI and alcoholic units per week was assessed through visual inspection of the histograms and presented as median with IQR due to skewness of the data. The proportion of individuals who had ever smoked was presented with corresponding 95% CI calculated using the Wald method.

### Role of the funding source

There was no funding source for this study.

## Results

**Figure 1:**
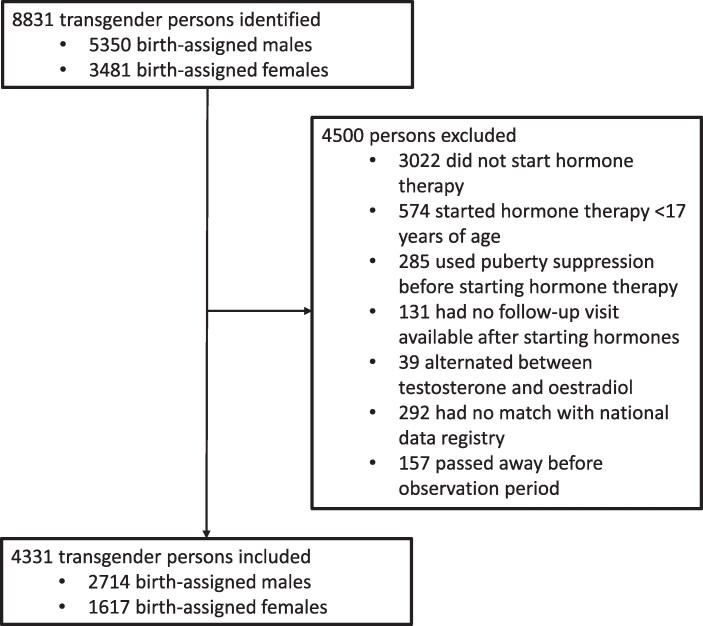
Study flowchart

The study cohort consists of 8831 persons (5350 birth-assigned males, 3481 birth-assigned females) who visited the gender identity clinic between 1972 and 2018. Four thousand five hundred were excluded for a variety of reasons. A total of 2714 transgender women and 1617 transgender men were included in the study (*Figure 1*). Characteristics of the study cohort are presented in *Table 1*. Median age at hormone therapy initiation was 30.5 (IQR 23.6–41.3) and 23.8 (IQR 20.3–31.7) years, respectively. The median and total follow-up time during the observation period were 10.9 (IQR 6.5–11.0) years and 23 907 person-years for transgender women and 8.4 (IQR 5.9–11.0) years and 13 457 person-years for transgender men. Two hundred thirty-two transgender women and 38 transgender men died during the observation period.

**Table 1:** Descriptive characteristics of the study cohort and cardiovascular event occurrence

	Transgender women (*N* = 2714)	Transgender men (*N* = 1617)
Age at start of hormone therapy (years)	30.5 (23.6–41.3)	23.8 (20.3–31.7)
Age at start of observation period (years)	42.9 (29.0–52.9)	28.5 (21.4–43.3)
Age at end of observation period (years)	52.5 (37.2–61.8)	36.7 (28.5–53.3)
Median follow-up during observation period (years)	10.9 (6.5–11.0)	8.4 (5.9–11.0)
Total time at risk during observation period (person-years)	23 907	13 457
Number of events during observation period (***n***, incidence rate per 1000 person-years and 95% CI)		
MI	31 (1.3/1000 person-years, 95% CI .9; 1.8)	25 (1.9/1000 person-years, 95% CI 1.2; 2.7)
CVA	74 (3.1/1000 person-years, 95% CI 2.4; 3.9)	28 (2.1/1000 person-years, 95% CI 1.4; 3.0)
VTE	57 (2.4/1000 person-years, 95% CI 1.8; 3.1)	17 (1.3/1000 person-years, 95% CI .7; 2.0)
Age at event (years)		
MI	57.8 (50.7–70.1)	58.4 (52.7–63.8)
CVA	59.6 (51.4–67.9)	52.0 (33.3–57.3)
VTE	59.3 (48.9–65.4)	45.3 (43.0–58.6)
Time between start of hormones and first event during observation period (years)		
MI	18.1 (5.9–26.2)	26.2 (18.2–30.1)
CVA	17.3 (8.0–26.6)	11.1 (6.4–24.5)
VTE	13.3 (6.0–26.2)	11.8 (6.0–24.3)

Data are presented as median (interquartile range), absolute numbers, and incidence rates (per 1000 person-years) with 95% confidence interval.

CI, confidence interval; MI, myocardial infarction; CVA, cerebrovascular accident; VTE, venous thromboembolism.

**Figure 2:**
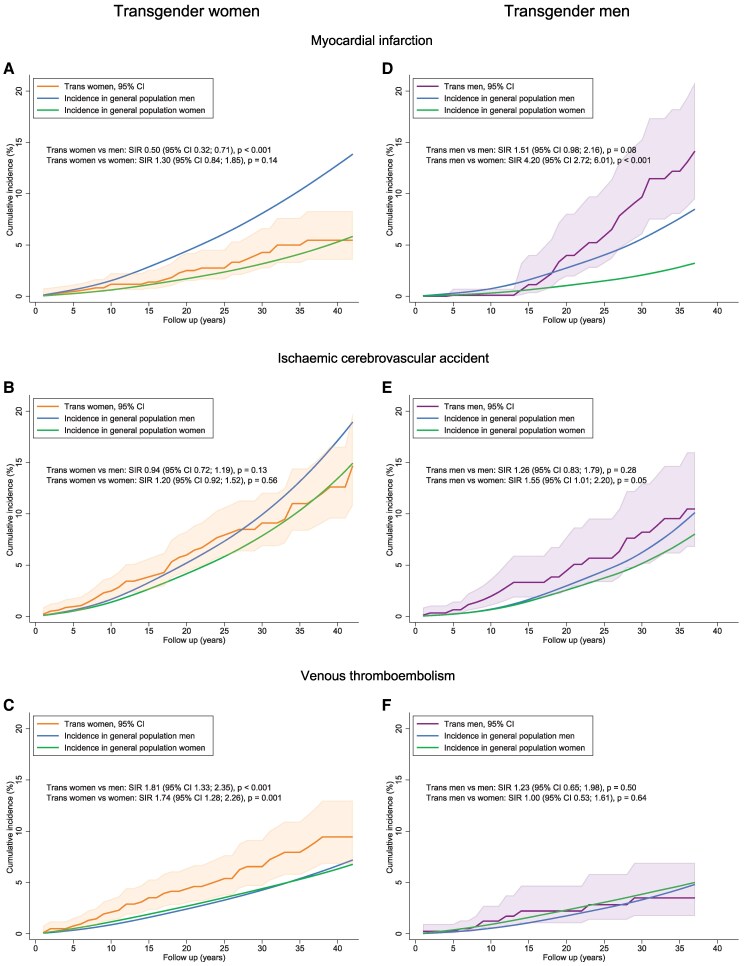
Cumulative incidence of acute cardiovascular events. For each year of follow-up, the observed cumulative incidence was set out against the expected cumulative incidence in the general population, derived from reference incidence rates of age-, sex-, calendar year-, and socioeconomic status-matched individuals from the general population. Standardized incidence ratios with 95% confidence intervals were calculated using an actuarial life table approach. Among 2714 transgender women, 31 myocardial infarctions, 74 cerebrovascular accidents, and 57 venous thromboembolisms were observed during the observation period. Among 1617 transgender men, 25 myocardial infarctions, 28 cerebrovascular accidents, and 17 venous thromboembolisms were observed

*Table 2* presents all crude and adjusted results from the comparative analyses of cardiovascular events. The adjusted results are visualized in *Figure 2*. During the observation period, transgender women experienced 31 MIs. Adjusted for SES, this was lower than expected compared with general population men (SIR .50 [95% CI .32; .71]) and similar compared with general population women (SIR 1.30 [95% CI .84; 1.85]). Cerebrovascular accident occurred in 74 transgender women, which was similar to general population men (SIR .94 [95% CI .72; 1.19]) and women (SIR 1.20 [95% CI .92; 1.52]). Venous thromboembolism was observed 57 times. This was more than expected compared with general population men (SIR 1.81 [95% CI 1.33; 2.35]) and women (SIR 1.74 [95% CI 1.28; 2.26]).

**Table 2:** Risk of acute cardiovascular events in transgender women and men compared with the general population

	Observed events (*n*)		Expected events (*n*)	SIR^a^ (age, sex, year) (95% CI)	SIR^b^ (age, sex, year, SES) (95% CI)
Transgender women (*N* = 2714)					
MI	31	vs general population women	19.8	**1.57** (1.06; 2.16, *P* = .03)	**1.30** (.84; 1.85, *P* = .14)
		vs general population men	52.0	**.60** (.40; .82, *P* < .001)	**.50** (.32; .71, *P* < .001)
CVA	74	vs general population women	56.4	**1.31** (1.03; 1.63, *P* = .04)	**1.20** (.92; 1.52, *P* = .56)
		vs general population men	72.4	**1.02** (.80; 1.27, *P* = .88)	**.94** (.72; 1.19, *P* = .13)
VTE	57	vs general population women	31.6	**1.80** (1.37; 2.30, *P* < .001)	**1.74** (1.28; 2.26, *P* = .001)
		vs general population men	32.6	**1.75** (1.33; 2.23, *P* < .001)	**1.81** (1.33; 2.35, *P* < .001)
Transgender men (*N* = 1617)					
MI	25	vs general population women	5.9	**4.25** (2.75; 6.06, *P* < .001)	**4.20** (2.72; 6.01, *P* < .001)
		vs general population men	16.6	**1.50** (.97; 2.15, *P* = .08)	**1.51** (.98; 2.16, *P* = .08)
CVA	28	vs general population women	17.4	**1.61** (1.07; 2.26, *P* = .04)	**1.55** (1.01; 2.20, *P* = .05)
		vs general population men	21.3	**1.32** (.87; 1.85, *P* = .21)	**1.26** (.83; 1.79, *P* = .28)
VTE	17	vs general population women	14.2	**1.20** (.70; 1.83, *P* = .56)	**1.00** (.53; 1.61, *P* = .64)
		vs general population men	12.2	**1.40** (.82; 2.14, *P* = .27)	**1.23** (.65; 1.98, *P* = .50)

Data are presented as absolute numbers and standardized incidence ratio with 95% confidence interval.

*P*-values are reported to two decimal places, except when *P* < .001 or *P* = .001.

MI, myocardial infarction; CVA, ischaemic cerebrovascular accident; VTE, venous thromboembolism; SIR, standardized incidence ratio; SES, socioeconomic status.

Standardized incidence ratios are shown in bold.

^a^Standardized incidence ratio standardized for age, sex, and calendar year.

^b^Standardized incidence ratio standardized for age, sex, calendar year, and socioeconomic status.

During the observation period, 25 transgender men experienced a MI, which was higher than expected compared with general population women (adjusted SIR 4.20 [95% CI 2.72; 6.01]) and also tended to be higher compared with general population men (adjusted SIR 1.51 [95% CI .98; 2.16]). Cerebrovascular accident was observed in 28 transgender men. Adjusted for SES, SIR compared with general population women was 1.55 (95% CI 1.01; 2.20) and compared with general population men 1.26 (95% CI .83; 1.79). Venous thromboembolism occurred 17 times, which was similar to general population women (SIR 1.00 [95% CI .53; 1.61]) and men (SIR 1.23 [95% CI .65; 1.98]).

**Figure 3:**
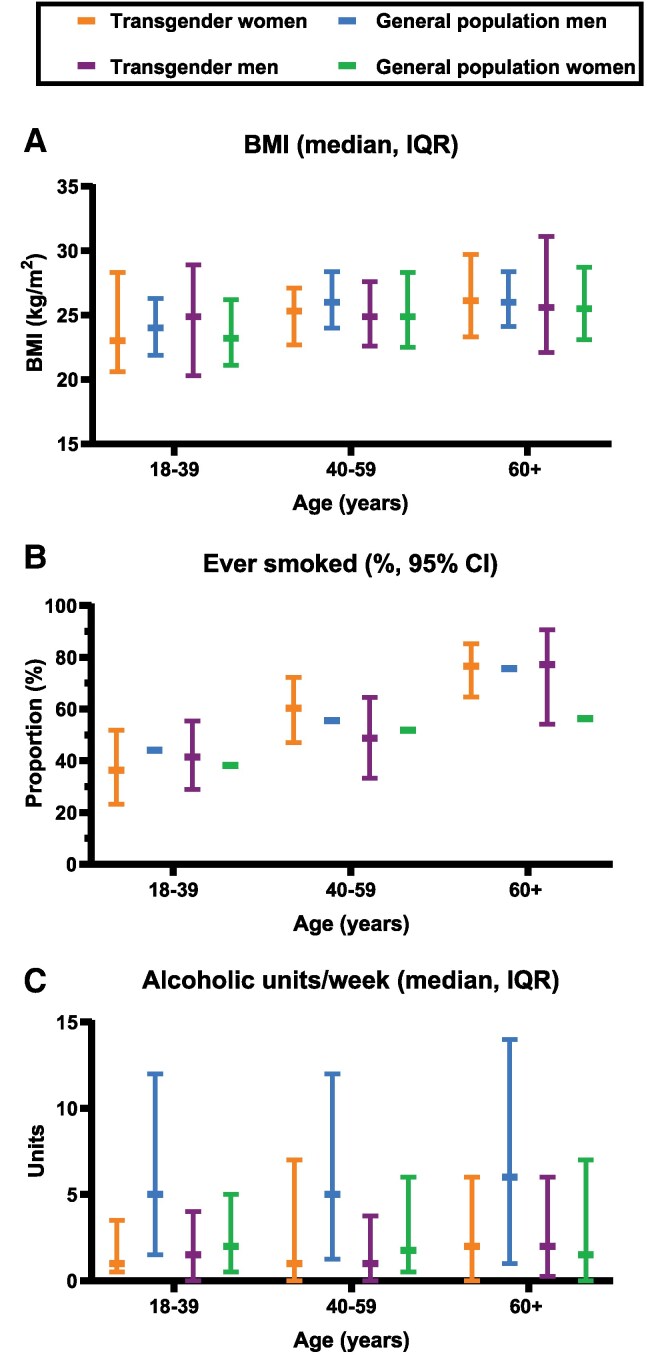
Descriptive results for body mass index, smoking history, and alcohol consumption stratified by age group. Results are presented as median and % (95% confidence interval). Results are based on data from 174 transgender women and 117 transgender men, as well as 731 411 men and 848 311 women from the general population

Statistics Netherlands provides lifestyle data based on extensive national surveys. Unfortunately, data were available for only 291 transgender persons (174 transgender women, 117 transgender men). Among the general population, data from 731 411 men and 848 311 women were available. Descriptive results are presented in *Figure 3*. Overall, lifestyle factors in transgender individuals were comparable to those in men and women from the general population within each age category. Only among individuals aged 60 years and older, a slightly higher proportion of transgender men had ever smoked (77.3% [95% CI 54.1; 90.7]) compared with women in the general population (56.4% [95% CI 56.2; 56.5]).

## Discussion

This study investigated the impact of GAHT on the risk of three major cardiovascular events in transgender persons by comparing incidence rates with those in the general population and accounting for SES and lifestyle. Contrary to prior research, our results suggest that oestradiol use lowers the risk of MI and does not affect CVA risk in transgender women. The higher risk of VTE remains. In transgender men, testosterone use is linked to a substantial increase in MI risk and, to a lesser extent, an increase in CVA risk, while it does not affect VTE risk (*Structured Graphical Abstract*).

Our findings in transgender women align with the established beneficial changes in blood pressure and lipid profile following the initiation of feminizing GAHT.^5–7^ Contrary to our expectations, SES had a relatively small impact on the risk of cardiovascular events in transgender women. The differences between our findings and those of previous studies are likely attributable to our use of a control population comprising all residents of the Netherlands, with data for the transgender and control groups obtained from the same national registry, rather than the use of a matched or selectively chosen reference group, with data collected from a different source than for the transgender population. This presumably resulted in a more representative estimation of cardiovascular risk in transgender individuals in our study. In addition, larger differences in SES and lifestyle disparities between transgender and cisgender persons may have contributed to the higher risks of MI and CVA found in transgender women in prior studies. Although we were unable to adjust for lifestyle due to data availability limitations, we explored potential differences in lifestyle factors between Dutch transgender persons and people from the general population in a subset of our study population. The descriptive results show minimal differences between transgender women and general population men, except for lower alcohol consumption in transgender women. However, higher smoking rates and obesity prevalence have been reported in other transgender populations, raising the question whether lifestyle disparities may have affected the results in previous studies.^21,22^ The elevated MI and CVA risk in transgender men compared with general population women is consistent with previous literature, although in our cohort, the risk for MI is considerably higher.^4,23^ Myocardial infarction risk is still substantially increased after adjusting for SES, suggesting that this is an effect of testosterone use. Besides blood pressure and lipid levels, testosterone leads to elevated haematocrit levels.^5–7^ Subsequently, erythrocytosis occurs in 11% of transgender men. In cisgender men, erythrocytosis increases the risk of cardiovascular events, but this association has yet to be investigated in transgender men.^24^ The similarity in lifestyle factors between transgender men and women in the general population, apart from a slightly higher prevalence of ever-smokers in the oldest age group, suggests that lifestyle differences are unlikely to explain the observed disparity in cardiovascular event risk.

Although transgender women do not face a higher risk of arterial cardiovascular events, their risk of VTE is elevated. Higher incidence of venous thrombosis has been consistently reported in previous studies on transgender women and is not surprising, given the well-established prothrombotic effects of oestradiol as observed in oral contraceptives and hormone replacement therapy for cisgender women.^4,25,26^ A similar VTE risk for transgender men and general population women was found, indicating that VTE risk is not affected by testosterone.

In addition to comparing transgender persons with people of the same sex assigned at birth, we also made comparisons based on their experienced gender. In transgender women compared with general population women, the risk of VTE remains elevated after adjustment for SES. This observation further indicates that GAHT leads to a shift in cardiovascular risk profile towards that of the experienced gender, which was already suggested in previous studies investigating changes in cardiometabolic parameters.^5–7^ In transgender men, the risk of MI tends to be higher not only compared with general population women but also with general population men. The underlying cause is unclear, but possible explanations include a potential effect of unmeasured confounders, the effect of exogenous testosterone on the body of someone who was assigned female at birth potentially differing from the effects of endogenous testosterone in cisgender men, or exposure to periods of supraphysiological doses of testosterone in transgender men, particularly when using short-acting testosterone esters.

Our study is the first to adjust the risk for cardiovascular events in transgender persons for SES based on three components: education level, recent employment history, and household income. This provides important insights, as previous studies have attributed the increased cardiovascular risk to the use of GAHT without considering the impact of SES.^27–29^ The small effect that SES has on cardiovascular risk in transgender men suggests that their socioeconomic position might be similar to that of cisgender peers. In contrast, SES did affect MI and CVA risk in transgender women, suggesting a poorer socioeconomic position in transgender women compared with people from the general Dutch population. It is important to notice that the socioeconomic position and its effect on cardiovascular health might play a larger role in other countries where transgender persons more frequently face severe social and institutional discrimination and where access to healthcare is less available or not publicly funded as opposed to the Netherlands and other Western European countries.^14,30,31^ This could partially explain the higher cardiovascular risk observed in transgender women in previous studies that did not account for SES. Surprisingly, a Danish study did not find SES to be a confounder for the risk of the outcome ‘any cardiovascular disease’ in transgender persons. In this study, SES was defined solely by personal income, and the transgender cohort also included persons who did not use GAHT.^30^ Instead, the authors suggested GAHT to be a mediator in the relationship between being transgender and the risk of cardiovascular disease. However, social and hormonal transition can affect SES, so GAHT should not be considered an independent risk factor for CVD, but evaluated within the broader context of the transition’s impact on socioeconomic position. This is illustrated by a Swedish study, where higher CVD rates in transgender persons compared with cisgender persons were found, but no differences between transgender persons who did and who did not use GAHT.^32^

The use of national registry data allowed us to include the entire Dutch population as reference, identify cardiovascular events without any loss to follow-up, and adjust for SES. By applying an actuarial life table approach, we addressed the limitation of being restricted to the observation period from 2012 to 2022 during which cardiovascular events were registered. This methodology addresses the limitations of a previous study on cardiovascular events by our research group, which used a Norwegian cohort as the control group, had a maximum follow-up until 2015, and did not have access to national registry data to collect outcome data in the transgender individuals.^23^ Cardiovascular events occurring after a person’s last hospital visit were not documented, resulting in incomplete outcome data in this previous study by Nota *et al*. For future meta-analytic purposes, the number of observed events from the overlapping observation periods from 2012 to 2015 is provided as Supplementary data online, *Table S1*. Notably, higher SIRs for transgender women were observed in the prior study by Nota *et al*. This may partly be explained by their use of a Norwegian control population, which may be different from the general Dutch population. Furthermore, in the study by Nota *et al*., outcomes were collected differently for transgender individuals (medical records) and controls (registry data), whereas in our study, outcome data for both groups were obtained from the same national registry. While the use of registry data ensured complete follow-up for outcome data for Dutch residents, the possibility of some loss to follow-up due to emigration cannot be entirely excluded. Markedly, Nota *et al*.’s study covers a period of time during which EE was commonly prescribed to transgender women, whereas in our study, we only observed events from 2012 onwards, by which time EE was no longer used in the Netherlands. Given EE’s higher thrombosis risk compared with other oestradiol forms, this may have contributed to the higher VTE SIRs in transgender women in the study by Nota *et al*. Although our analyses accounted for age, sex, calendar year, and SES by including these variables as stratification factors, a major limitation of our study is the presence of residual confounding, as we were unable to stratify for other important confounders, such as lifestyle factors, minority stress, mental health problems, and the use of certain psychotropic medications due data unavailability. Nevertheless, we explored the potential impact of lifestyle using the available data. Residual confounding may also have occurred due to measurement error in SES, for example, in cases of unregistered labour or undeclared income, and the categorization of SES into tertiles may have led to a loss of information and reduced precision. Another limitation is that, within this observational study, not all assumption underlying the applied cause-specific approach for handling competing events could be fully met. For example, the competing event of death from any cause cannot be prevented through a single, well-defined intervention, and unmeasured confounding cannot be ruled out. Therefore, the estimated SIRs should be interpreted with appropriate caution. Furthermore, changes in hormone modalities over the years complicate investigating the specific impact of different hormone regimens on the risk of cardiovascular events. A sensitivity analysis in transgender women having used EE or cyproterone acetate in the past was not performed, because almost all transgender women who started GAHT before 2001 and 2018, respectively, have used these types of hormones. The time at risk to develop cardiovascular events is therefore still too short for the majority of transgender women who started GAHT more recently. We hope to study this in the future, when transgender women who started GAHT more recently have reached longer follow-up periods. In transgender men, use of intramuscular testosterone may lead to a different risk of cardiovascular disease than testosterone gel, possibly because of higher peak testosterone levels after short-acting injections. However, we were not able to study this with the available data.

In conclusion, our findings indicate that GAHT is associated with a reduced MI risk in transgender women, as well as a substantially increased MI risk in transgender men. The impact of GAHT on CVA risk appears to be less pronounced in both groups. However, GAHT is associated with an increased VTE risk exclusively in transgender women. Our results provide insights into a longstanding paradox by demonstrating that GAHT does not reverse traditional sex differences in cardiovascular disease patterns, but instead aligns cardiovascular risk in transgender people with the risk profiles associated with the specific hormones that are used.

## Supplementary Material

ehaf837_Supplementary_Data

## Declarations

### Data Availability

The results in this study are based on calculations by the authors using non-public microdata from Statistics Netherlands. To ensure the anonymity of individuals in their databases, Statistics Netherlands does not allow the sharing of data. However, under certain conditions, these microdata are accessible for statistical and scientific research. For further information, please contact microdata@cbs.nl.
