# Supplementary material for: Transgender persons receiving gender-affirming hormone therapy: risk of acute cardiovascular events in a Dutch cohort study
Source: Eur Heart J. 2025 Nov 4;47(21):2660–9. doi: 10.1093/eurheartj/ehaf837 (PMC13225882; doi:10.1093/eurheartj/ehaf837)
Supplement: ehaf837_Supplementary_Data [file ehaf837_supplementary_data.docx]

**Supplemental material**

Table S1. Number of observed events per time period for the current study and a study previously conducted by our research group (19). This table shows that our current study was able to identify more events because of the link with the national data registry Statistics Netherlands.

|  |  | **MI (n)** | | | **CVA (n)*** | | | **VTE (n)** | | |
| --- | --- | --- | --- | --- | --- | --- | --- | --- | --- | --- |
|  | **Period** | **1972-2011** | **2012-2015** | **2016-2022** | **1972-2012** | **2012-2015** | **2016-2022** | **1972-2012** | **2012-2015** | **2016-2022** |
| **Current study** | **Trans women**  **(N = 2714)** | NA | 12 | 19 | NA | 22 | 52 | NA | 12 | 45 |
|  | **Trans**  **men**  **(N = 1617)** | NA | 3 | 22 | NA | 6 | 22 | NA | 3 | 14 |
| **Nota et al. (2019)** | **Trans women**  **(N = 2517)** | 24 | 6 | NA | 17 | 4 | NA | 70 | 3 | NA |
|  | **Trans men**  **(N = 1358)** | 10 | 1 | NA | 6 | 0 | NA | 1 | 1 | NA |

MI: myocardial infarction. CVA: ischemic cerebrovascular accident. VTE: venous thromboembolism. NA: not applicable. *In the study by Nota et al. (19), CVA also included hemorrhagic stroke and subarachnoid hemorrhage. However, to enable comparison of the observed events, this table only presents the number of ischemic strokes and transient ischemic attacks.
